# Management of LDL‐cholesterol after an acute coronary syndrome: Key comparisons of the American and European clinical guidelines to the attention of the healthcare providers

**DOI:** 10.1002/clc.23410

**Published:** 2020-06-29

**Authors:** Baris Gencer, Robert P. Giugliano

**Affiliations:** ^1^ TIMI Study Group, Division of Cardiovascular Medicine, Brigham and Women's Hospital Harvard Medical School Boston Massachusetts USA; ^2^ Cardiology Division Geneva University Hospitals Geneva Switzerland

**Keywords:** ezetimibe, guideline, lipids, PCSK9 inhibitors, prevention, statin

## Abstract

Guidelines for the management of blood cholesterol were updated in the past year in the United States and Europe, reflecting a more intensive approach to lowering low‐density lipoprotein cholesterol (LDL‐C). The American College of Cardiology/American Heart Association task force on practice guideline released the 2018 guideline on the management of blood cholesterol on behalf of several American societies. Approximately 9 months later, the European Society of Cardiology/European Atherosclerosis Society published their 2019 guideline for the management of dyslipidemias. Both guidelines have similarities for the management of patients with acute coronary syndromes. Both emphasize risk assessment of patients as a main approach to guide therapy; those at higher risk of cardiovascular disease have a greater clinical benefit of LDL‐C reduction by at least 50%. Both guidelines reinforce the indication to lower LDL‐C as an important modifiable risk factor and consider the addition of nonstatin agents, such as ezetimibe and proprotein convertase subtilisin kexin 9 (PCSK9) inhibitors, in addition to lifestyle counseling and high‐intensity statin for further reduction of LDL‐C levels. However, the guidelines have differences in the concepts of treatment thresholds (≥70 mg/dL in the United States) vs treatment goals (< 55 mg/dL in Europe), in the definition of very high‐risk category and in the classes for recommendation for the use of PCSK9 inhibitors.

## 1 INTRODUCTION

Guidelines for the management of blood cholesterol were updated in the past year in the United States and Europe, reflecting a more aggressive approach to lowering low‐density lipoprotein cholesterol (LDL‐C).^1^, ^2^ The American College of Cardiology/American Heart Association (ACC/AHA) task force on practice guideline released the 2018 guideline on the management of blood cholesterol on behalf of several American societies.^1^ Approximately 9 months later, the European Society of Cardiology (ESC)/European Atherosclerosis Society published their 2019 guideline for the management of dyslipidemias: lipid modification to reduce cardiovascular risk.^2^ The main document for the US guideline consists of 69 pages (bibliography and appendix not included), 282 references including those presented in the supplemental materials and 72 recommendations, including 29 of class I (40.3%), 25 of class IIa (34.7%), 15 of class IIb (20.9%), and 3 of class III (4.2%).^1^ The US guidelines was based on an independent systematic evidence review. The main document for the European guideline was slightly shorter (59 pages) with more references (608) and a similar number of recommendations (69), including 36 of class I (52.2%), 18 of class IIa (26.1%), 10 of class IIb (14.5%), and 5 of class III (7.2%).^2^

The ability to reach low LDL‐C with novel therapies and studies in patients with genetic variants resulting in very low LDL‐C levels has dramatically changed lipid management. Guideline recommendations regarding the treatment goals for LDL‐C in high or very high‐risk patients have plummeted from 130 mg/dL (3.4 mmol/L) in 1988 to 55 mg/dL (1.4 mmol/L) in 2019.^1^, ^3^, ^4^ The scientific evidence of lowering LDL‐C levels in patients after acute coronary syndromes (ACS) is principally based on five adequately powered randomized controlled trials: the Myocardial Ischemia Reduction with Aggressive Cholesterol Lowering study (atorvastatin 80 mg/day vs placebo),^5^ the Pravastatin or Atorvastatin Evaluation and Infection Therapy‐Thrombolysis in Myocardial Infarction 22 study (atorvastatin 80 mg/day vs pravastatin 40 mg/day),^6^ the Early Intensive vs a Delayed Conservative Simvastatin Strategy in Patients With Acute Coronary Syndromes (phase Z of the A to Z Trial), study (simvastatin 40 mg/day vs placebo, then simvastatin 80 mg/day vs simvastatin 20 mg/day),^7^ the Improved Reduction of Outcomes: Vytorin Efficacy International Trial,^8^ and the Evaluation of Cardiovascular Outcomes After an Acute Coronary Syndrome During Treatment With Alirocumab study (alirocumab vs standard care).^9^ To this, we can add the recent myocardial infarction subgroup analysis from the Further Cardiovascular Outcomes Research With PCSK9 Inhibition in Subjects With Elevated Risk (FOURIER) study (evolocumab vs standard care).^10^, ^21^

**FIGURE 1:**
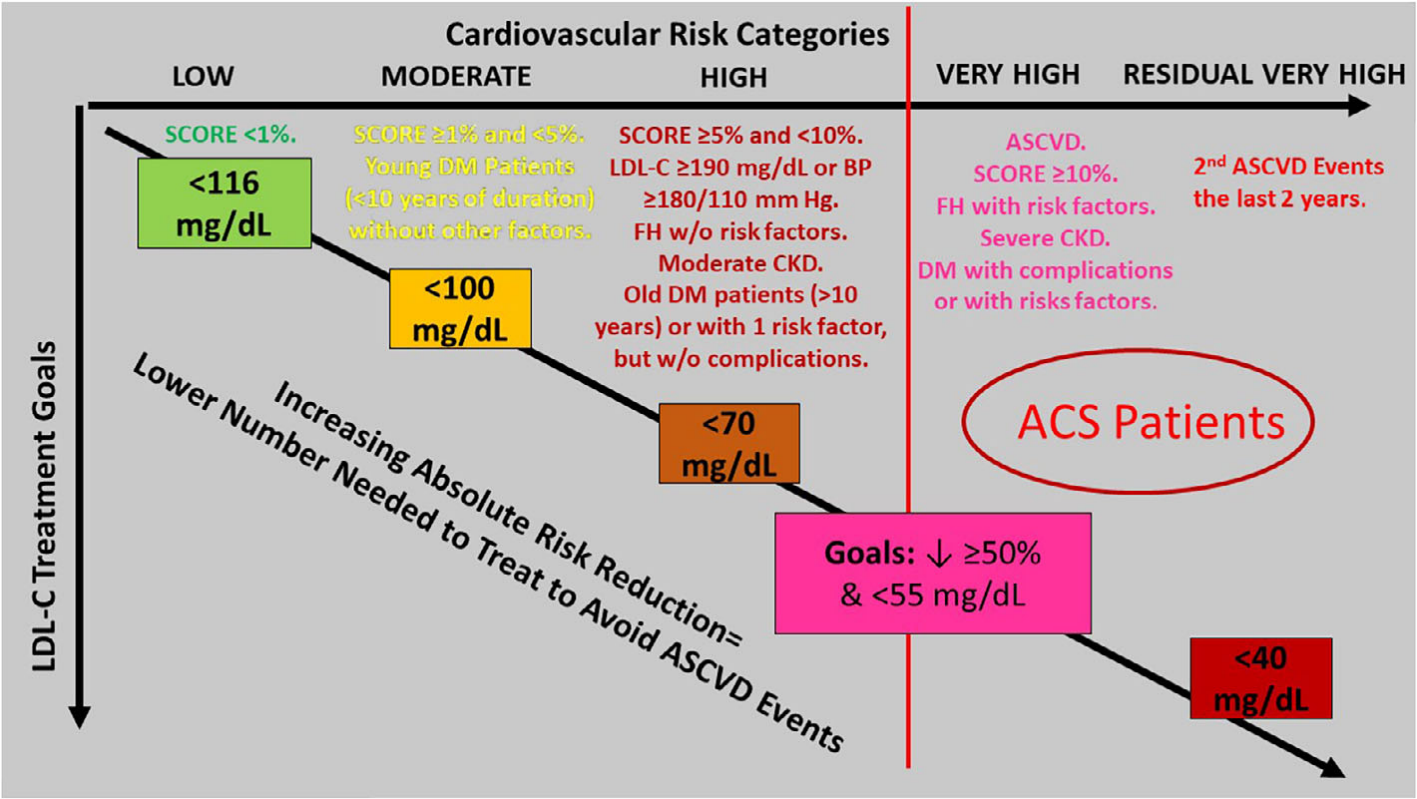
Risk stratification and LDL‐C targets as recommended by the European guideline for the management of dyslipidaemias.^2^ ACS, acute coronary syndromes; ASCVD, atherosclerotic cardiovascular disease; BP, blood pressure; CKD, chronic kidney disease; DM, diabetes mellitus; FH, familial hypercholesterolemia; LDL‐C, low‐density lipoprotein cholesterol; DM, diabetes mellitus; SCORE: Systematic Coronary Risk Estimation

**FIGURE 2:**
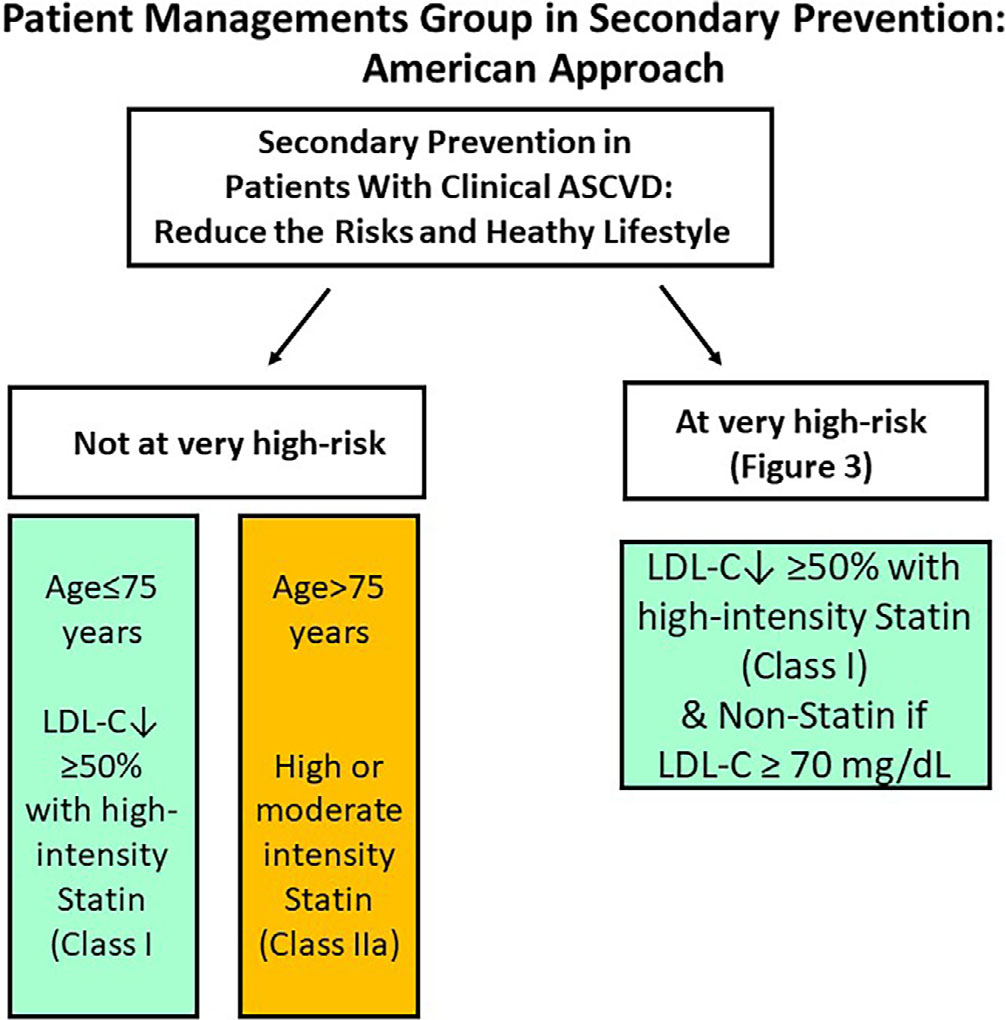
Patient management groups as recommended by the American guideline for the management of cholesterol.^1^ Figure adopted from ref.^1^ LDL‐C, low‐density lipoprotein cholesterol

The guidelines have many similarities. Both emphasize cardiovascular (CVD) risk assessment of patients as a main approach to guide therapy (Figures 1, 2). Both guidelines reinforce the indication to lower LDL‐C as an important modifiable risk factor and consider the addition of nonstatin agents, such as ezetimibe and proprotein convertase subtilisin kexin 9 (PCSK9) inhibitors, in addition to lifestyle counseling and high‐intensity statin for further reduction of LDL‐C levels.^1^, ^2^ Both guidelines have the potential to change the current practice, as the use of moderate dose of statin is still frequent in a large number of patients after ACS and unlikely to be sufficient to reduce efficiently the cardiovascular risk.^11^, ^12^

However, the guidelines have important differences, including the concepts of treatment thresholds (American) vs treatment goals (European) and the specific classes for recommendation most notably in secondary prevention (Table 1). In this manuscript, we summarize the key message of both guidelines focusing on the management of cholesterol in patients after ACS.

**TABLE 1:** Comparison of the treatment strategies in patients with ACS as recommended by the American and European cholesterol guidelines

	American guideline^1^	European guideline^2^
Thresholds vs goals in ACS patients	Use a maximal statin to lower LDL‐C levels by ≥50% (class I) after ACS. Use a threshold of ≥70 mg/dL to consider addition of non‐statins to statin therapy after ACS (class IIa). Concept of on LDL‐C threshold aims to reduce risk by lowering further LDL‐C when the values are above criteria used in non‐statin RCTs. Strategies using LDL‐C threshold have been examined in RCTs.	LDL‐goals in ACS patients are both a reduction of ≥50% and < 55 mg/dL (class I). LDL‐goal in recurrent ASCVD events within 2 years is <40 mg/dL (class IIb). Approaches with LDL‐C goals aim to reduce risks by lowering LDL‐C to levels achieved in large RCTs. Strategies using goals have rarely been evaluated in RCTs.
Treatment algorithms in ACS patients	1.Healthy lifestyle (class I). 2.High‐intensity maximal statin in combination with ezetimibe if PCSK9‐I considered (class I). 3. If LDL‐C ≥ 70 mg/dL within 4‐12 weeks, reasonable to add PCSK9‐i (class IIa).	1.Healthy Lifestyle (class I). 2.High‐intensity maximal statin (class I). 3.If LDL‐C ≥ 55 mg/dL within 4‐6 weeks, add ezetimibe (class I). 4.If LDL‐C ≥ 55 mg/dL within 4‐6 weeks, add PCSK9‐i (class I). 5. If LDL‐C ≥ 40 mg/dL within 4‐6 weeks and recurrent ASCVD event within 2 years, may add PCSK9‐I (class IIb).

Abbreviations: ACS, acute coronary syndromes; LDL‐C, low‐density lipoprotein cholesterol; PCSK9, proprotein convertase subtilisin kexin 9; RCT, randomized controlled trials.

## 2 SECONDARY PREVENTION RISK STRATIFICATION

### 2.1 General comments

**FIGURE 3:**
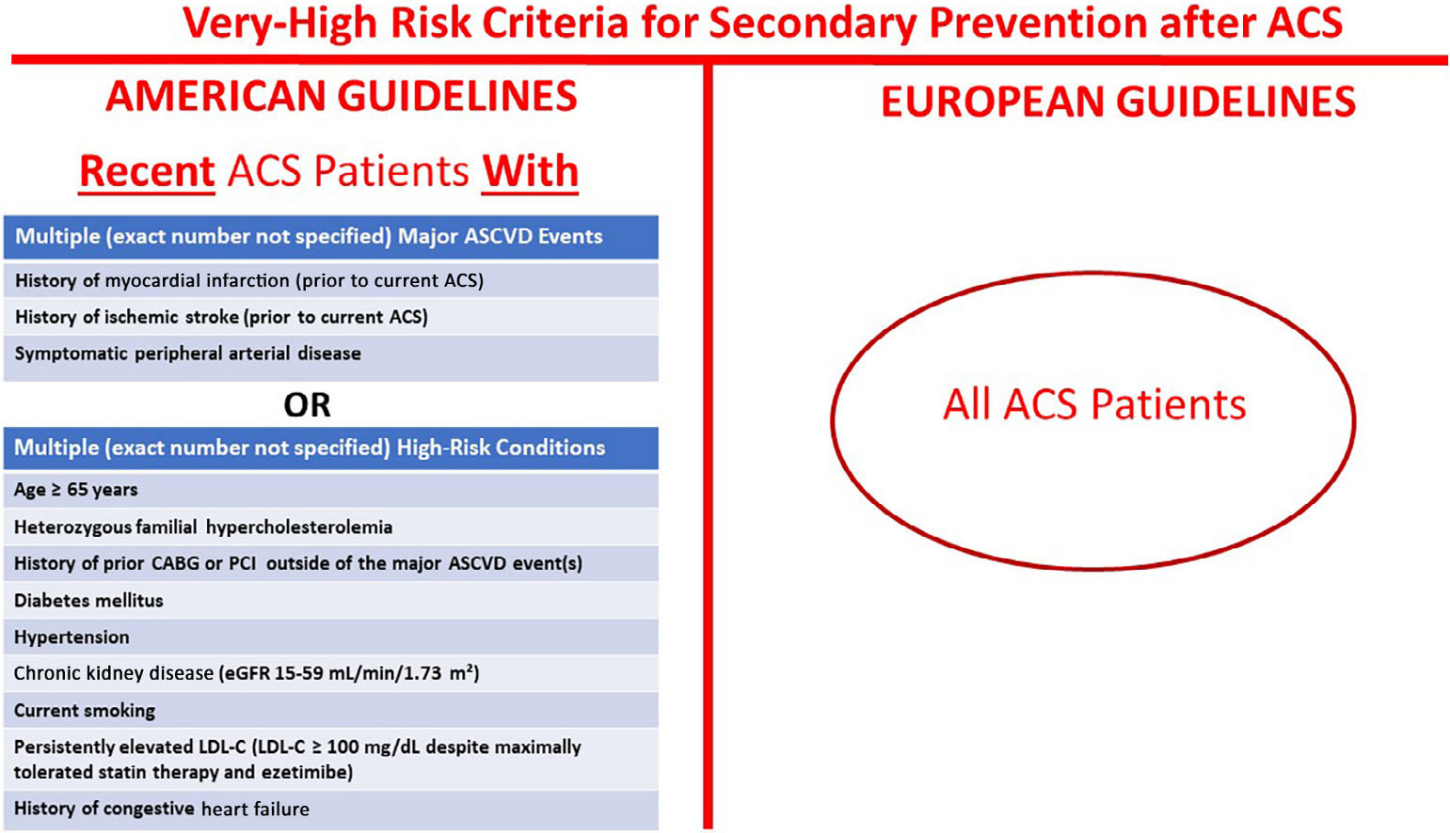
Differences in risk stratification for patients with acute coronary syndromes as recommended by the American guideline for the management of cholesterol and European guideline for the management of dyslipidaemias.^1^, ^2^ ACS, acute coronary syndromes; ASCVD, atherosclerotic cardiovascular disease; CABG, coronary artery bypass graft; LDL‐C, low‐density lipoprotein cholesterol; PCI, percutaneous coronary intervention

In the European guideline, all patients with an ACS are classified as very high risk, whereas in American guideline, a patient with ACS must also have multiple high‐risk features or more than one previous atherosclerotic cardiovascular disease (ASCVD) event (Figure 3).

### 2.2 American perspectives

The 2018 ACC/AHA guideline defines patient management groups of risk with specific algorithms for treatment (Figure 2). Secondary prevention for ASCVD is indicated in patients with a history of ACS (unstable angina or MI), stable angina or coronary revascularization, stroke, transient ischemic attack (TIA), or peripheral artery disease including aortic aneurysm. Among the patients with established ASCVD, very high‐risk patients were defined by the presence of multiple major ASCVD events (recent ACS plus another event) or one major (recent ACS) and multiple high‐risk conditions (Figure 3).

### 2.3 European perspectives

The 2019 ESC guideline considers all patients with previous ACS (MI or unstable angina) as very high‐risk (Figure 3). Other patients with established ASCVD at very high‐risk included patients with stable angina, and those with previous documented coronary revascularization, stroke, TIA, or peripheral artery disease (Figure 1).^2^

## 3 LDL‐C GOALS VS THRESHOLD FOR TREATMENT

### 3.1 General comments

For patients with ACS, the European guideline recommends a LDL‐C goal of <55 mg/dL and a LDL‐C reduction by 50% (class I) to guide therapy, whereas the American guideline recommends a high‐intensity statin to achieve a 50% reduction in LDL‐C and a threshold of ≥70 mg/dL for treatment intensification (Table 1).^1^ The approach with goals aims to reduce risks by lowering LDL‐C to levels achieved in large clinical trials, whereas the approach with threshold aims to reduce risks by lowering further LDL‐C when the LDL‐C values are above criteria used in nonstatin trials.^1^, ^2^ Threshold has been evaluated in many randomized clinical trials, with the baseline LDL‐C level predefined by the protocol inclusion criterion. Treatment goals have rarely been studied in randomized trials; therefore, the evidence is weaker and mostly based on postrandomization data (inference based on achieved LDL‐C levels by assigned treatment). The hazards of drawing medical decision from postrandomization data are still a matter of debate, although the data show consistent benefits of reducing LDL‐C below guideline recommendations.^13^, ^15^

### 3.2 American perspectives

In secondary prevention of patients at very high‐risk for ASCVD, the initiation or continuation of high‐intensity statin is recommended to achieve an LDL‐C reduction of ≥50%.^1^ If LDL‐C levels remain above the threshold of ≥70 mg/dL despite maximally tolerated therapy, the guideline recommends adding a nonstatin agent. If the LDL‐C is already <70 mg/dL, the continuation of the maximally tolerated therapy is recommended with monitoring of LDL‐C. The concept of threshold utilizes an adaptive strategy (ie, addition of nonstatin agent) only if the treatment effect observed with a standard therapy is not satisfactory (eg, LDL‐C levels on maximally tolerated statin still above the threshold). Therefore, once the LDL‐C is below the threshold of 70 mg/dL, the exact value of LDL‐C does not influence further modification of LDL‐lowering therapy (eg, having a value of 30 mg/dL is managed the same as a value of 68 mg/dL).

### 3.3 European perspectives

In secondary prevention of very high‐risk patients, an LDL‐C reduction of ≥50% from baseline and an LDL‐C goal <55 mg/dL are both recommended (class I, level A). A reduction in LDL‐C by 50% from baseline with high‐intensity statin is a first common step in both guidelines. However, European guideline recommends intensifying lipid‐lowering treatment even in patients who achieve on LDL‐C < 70 mg/dL, whereas the addition of nonstatin agent is recommended in the US guideline only for those above the threshold ≥70 mg/dL. In the European guidelines, patients with ASCVD who experienced a second vascular event within 2 years while taking maximally statin therapy, an LDL‐C goal of <40 mg/dL (<1 mmol/L) may be considered (class IIb, level B). Compared to the US guideline, the European guideline supports even lower LDL‐C goals with further intensification of therapy in patients with a recurrent ASCVD event with an LDL‐C of 55 to 70 mg/dL. The concept of goals implies that therapeutic options will be added sequentially to achieve a specific recommended LDL‐C level.

## 4 PHARMACOLOGICAL THERAPIES

### 4.1 General comments

For patients with ACS, the European guideline recommends the addition of nonstatin agents to high‐intensity maximal statin in a sequential approach (first ezetimibe, then PCSK9 inhibitors; both are class I recommendations) if the LDL‐C is above the target of >55 mg/L (Table 1) at each step. Whereas, the American guideline recommends the addition of ezetimibe (class I) to high‐intensity statin (sequential approach not specified) if the addition of PCSK9 inhibitors is considered. This is not deemed mandatory, but instead considered reasonable if the LDL‐C is above the threshold of ≥70 mg/dL (class IIa).

### 4.2 American perspectives

In secondary prevention of patients at very high‐risk, the use of ezetimibe is recommended if the LDL levels remain ≥70 mg/dL on maximally tolerated statin (class IIa).^8^ However, the addition of ezetimibe is recommended to maximally tolerated statin therapy as the first step in lowering LDL‐C (class I) if a combination with PCSK9 inhibitor as a third agent is being considered. The strategy of ezetimibe before PCSK9 inhibitor is recommended because generic ezetimibe is available, simple to administer (oral, once daily), and has proven safety and tolerability with long‐term data.^8^ Adherence to changes in lifestyle and effects of LDL‐C‐lowering medication should be assessed 4 to 12 weeks after statin initiation or dose adjustment and every 3 to 12 months thereafter for adherence and safety.^1^ If tolerated maximal LDL‐C lowering therapy, the LDL‐C ≥ 70 mg/dL or non‐HDL‐C is ≥100 mg/dL, the addition of PCSK9 is considered reasonable (class IIa).^9^, ^15^ The rationale regarding the recommendation for PCSK9 inhibitors in the US guidelines included cost considerations and a dedicated cost‐effectiveness analysis for the US population 1 in addition to the scientific evidence from the large clinical trials.^9^, ^10^

### 4.3 European perspectives

In patients after ACS, the guideline recommends starting high‐intensity statin or the maximum tolerated dose as early as possible, and regardless of initial LDL‐C values, to reach the goals for the very high‐risk group of ≥50% reduction from baseline and a goal of <55 mg/dL (class I, level A).^16^ Lipid levels should be evaluated 4 to 6 weeks after ACS to determine whether a reduction of ≥50% from baseline and an LDL‐C goal <55 mg/dL have been achieved. If the goal of <55 mg/dL is not achieved with the maximum tolerated dose of statin, combination with ezetimibe is recommended (class I, level B).^8^ If the goal <55 mg/dL is still not achieved on a maximum tolerated dose of a statin and ezetimibe after 4 to 6 weeks, a combination with a PCSK9 inhibitor is recommended (class I, level A).^9^, ^15^ For patients who present with an ACS and whose LDL‐C levels are not at goal, despite already taking a maximally tolerated statin dose and ezetimibe, the addition of a PCSK9 inhibitor early after the event (including prior to discharge) should be considered (class II, level C).^2^, ^17^

## 5 CLINICAL SCENARIO AND IMPLICATIONS

A 60‐years‐old patient with a history of smoking and diabetes is admitted with an NSTEMI. The angiography showed a significant lesion (90%) of right coronary artery that was revascularized successfully with angioplasty and drug eluting stent. This patient has additional nonobstructive lesions in the left anterior descending and left circumflex arteries, and the left ventricular ejection fraction is preserved. The LDL‐C levels obtained at hospital are 175 mg/dL. The initial management of both guidelines recommends healthy lifestyle and intensive maximal statin therapy.

Using the US guideline, we must first define whether this patient is at very high‐risk or not. This patient would be considered very high‐risk as the patient had ACS within 12 months and there are two high‐risk conditions (smoking and diabetes). High‐intensity statins should be started prior to discharge and the addition of ezetimibe considered early in the process (and before starting a PCSK9 inhibitor). In this case, if the LDL‐C level at 4 weeks after discharge was 61 mg/dL and below the threshold of 70 mg/dL, the therapy of high‐intensity statin and ezetimibe would be continued without modification until the next measurement in 3 to 12 months.

**FIGURE 4:**
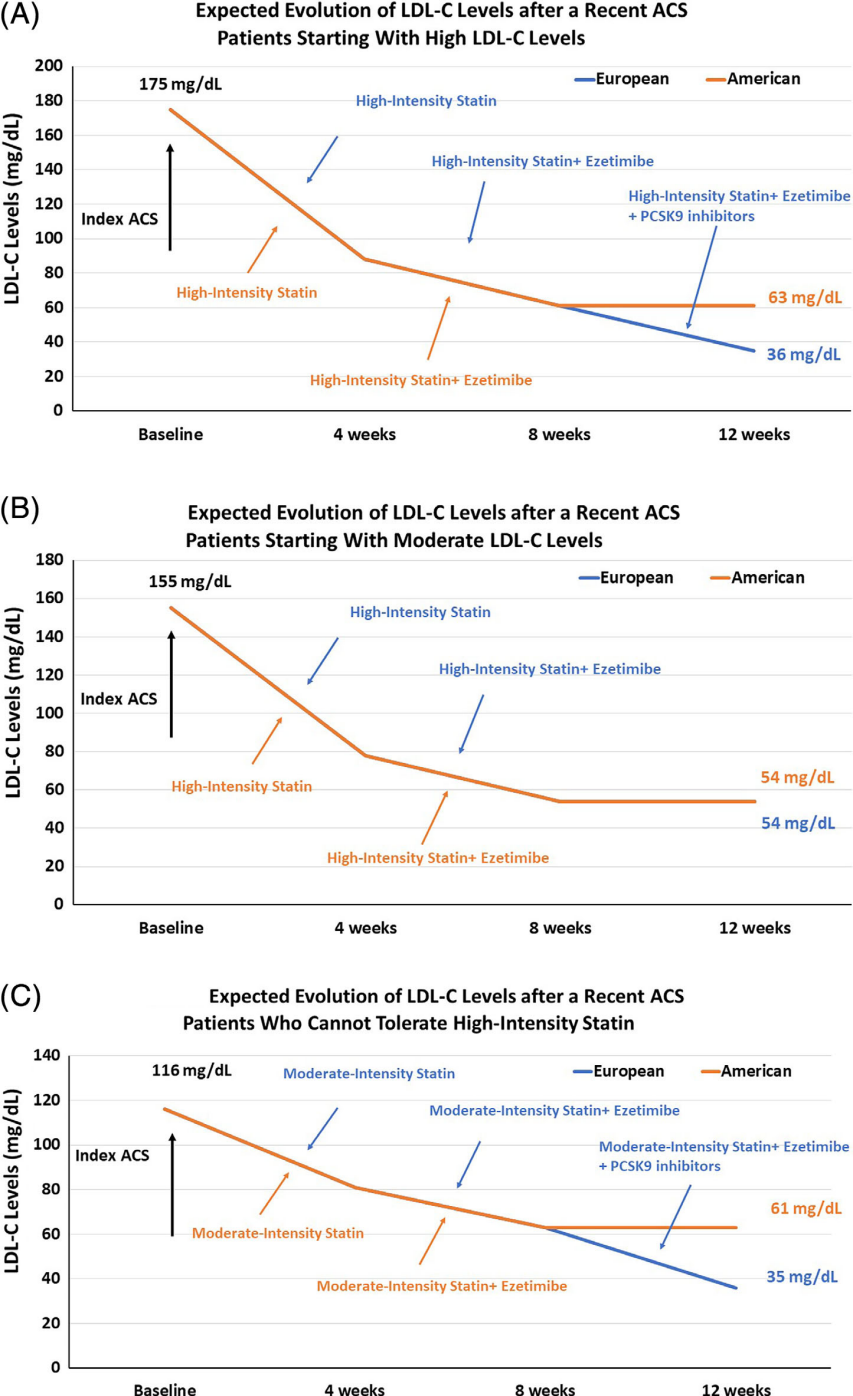
Changes in LDL‐C control in patients with high LDL‐C levels and recent acute coronary syndromes as recommended by the American guideline for the management of cholesterol and European guideline for the management of dyslipidemias. Panel A, Patients starting with high LDL‐C levels. Panel B, Patients starting with moderate LDL‐C levels. Panel C, Patients who cannot tolerate high‐intensity statin. ACS, acute coronary syndromes; LDL‐C, low‐density lipoprotein cholesterol; PCSK9, proprotein convertase subtilisin kexin 9

European guideline considers ACS automatically as a very high‐risk. Therefore, the addition of nonstatin agent to the maximally tolerated statin therapy can be considered if the LDL‐C level is ≥55 mg/dL. The combination of ezetimibe should be started only after 4 weeks after high‐intensity statin. Finally, if the LDL‐C after 8 weeks was 61 mg/dL on the combination of high‐intensity and ezetimibe, the target of ≤1.4 mmol/L would not have been achieved, and the addition of a PCSK9 inhibitor would be recommended. Finally, the LDL‐C after the introduction of a PCSK9 inhibitors reached the value of 35 mg/dL (in comparison with the value of 61 mg/dL when applying US guidelines, Figure 4 Panel A). This case illustrates a clinical scenario where the starting LDL‐C level is very high. For patients with lower starting LDL‐C level (< 155 mg/dL) who tolerate well the high‐intensity regimen of statin and ezetimibe, the on‐treatment LDL‐C level of 55 mg/dL is expected to be reached in the majority of patients without the need of PCSK9 inhibitors (Figure 4 Panel B). Finally, if we consider a third scenario with a patient starting with an LDL‐C of 116 mg/dL but who cannot tolerate high‐intensity statin, the combination of moderate‐intensity and ezetimibe will reduce the levels of LDL‐C to 63 mg/dL. With this on‐treatment level of LDL‐C, the patient would meet criteria for adding a PCSK9 inhibitor according to the European guideline, but not according to the American guideline (Figure 4 Panel C).

As highlighted above, the medical decision in some cases can differ when following recommendations of the American or European guidelines. The American guideline remains more conservative regarding the introduction of PCSK9 inhibitors as the treatment thresholds are less stringent (ie, intensification of therapy is triggered at a higher LDL‐C level). As specified in the preamble of the European guideline, cost was not a consideration in the recommendations. The European recommendations were based on achieved LDL‐C levels observed in the clinical trials and not based on LDL‐C level at the time of inclusion into the trial. This implies that application of European guideline in clinical practice would result in greater eligibility of PCSK9 inhibitors when compared with the American guideline. Neither the American nor the European guidelines mentioned that the addition of nonstatin agent to a standard high‐intensive statin therapy did not robustly improve life expectancy in patients with stable coronary heart disease. Further studies should evaluate the impact of the implementation of both guidelines on the control of LDL‐C levels, utilization of process outcome (eg, pattern of lipid‐lowering therapies prescription), clinical outcomes reduction, and cost‐effectiveness. In addition, new data with nonstatin agents (eg, icosapent ethyl and bempadoic acid),^18^, ^19^ and with the longer‐acting PCSK9 inhibitor inclisiran recently published will need to be considered in future guidelines.^20^

## 6 PERSPECTIVES

An alternative to the publication of multiple guidelines from various medical societies would be to develop a core document endorsed jointly across geographic regions, with specific sections that address specific regional issues. Such a process could streamline the numerous efforts made by the different societies and have a broader global impact on clinical practice and improvement of care, while permitting flexibility for national society to adapt the main guidelines based on the local healthcare system. It remains debatable whether medical guidelines should focus on summarizing the best available evidence to improve the care of the patients in an ideal world or also include additional health economic considerations to make the recommendations more realistic and appropriate for the clinicians. These distinctive approaches explain some of the differences between the American and European guideline for the management of cholesterol and the use of PCSK9 inhibitors.

## 7 LIMITATIONS

As limitations, none of the guidelines mentioned the optimal duration of pursuing high‐intensity lipid‐lowering therapies with the addition of PCSK9 inhibitors. The use of electronic health records, insurance claims data, and post‐marketing open‐label registries can supplement data from randomized trials regarding the long‐term tolerability and sustainability of lipid‐lowering therapies in clinical practice. In clinical trials, the use of PCSK9 inhibitors in patients with recent myocardial infarction demonstrated a benefit over a treatment period of 3 years,^21^ without major adverse events, even in those who attained very low LDL‐C levels with evolocumab (< 20 mg/dL).^15^, ^22^ Similar findings were reported among patients in patients achieving an LDL‐C < 30 mg/dL with statin plus ezetimibe and following for 6 years.^13^ Finally, we acknowledge that the cost‐effectiveness remains an important issue for PCSK9 inhibitors. Since the current guidelines recommendations for nonstatin focused on a very high‐risk group of patients, the cost‐benefit ratio of PCSK9 inhibitors should be more favorable in recommended patients compared to subjects at lower risk of CV complications. Providing access to those who would benefit most, regardless of socioeconomic or other barriers to guideline‐directed care, remains a challenge for newer more expensive therapies.

## 8 CONCLUSION

The benefit of intensive LDL‐C lowering to reduce cardiovascular risk is recognized in both guidelines in patients after an ACS. The risk reduction management of patients with ACS is based on adapting lipid‐lowering therapies according to the recommended treatment effect on LDL‐C levels and patients' characteristics.

## CONFLICT OF INTEREST

Dr G. B. activities in the TIMI Group, Harvard Medical Schools, are supported by grants from the Geneva University Hospitals, Eugenio Litta, and Arthemis Foundations. Dr. R.P. G report that his institution received research grant support from Amgen for his role as a member of the Executive Committees of the FOURIER, FOURIER‐LEGACY, and VESALIUS‐CV trials, and that he has received honoraria for CME lectures and/or consulting from Akcea, Amgen, Amarin, the American College of Cardiology, Bristol Myers Squibb, CVS Caremark, Daiichi Sankyo, GlaxoSmithKline, Merck, Pfizer, and Sanofi.
